# Whole genome-based characterisation of antimicrobial resistance and genetic diversity in *Campylobacter jejuni* and *Campylobacter coli* from ruminants

**DOI:** 10.1038/s41598-021-88318-0

**Published:** 2021-04-26

**Authors:** Medelin Ocejo, Beatriz Oporto, José Luis Lavín, Ana Hurtado

**Affiliations:** 1 Animal Health Department, NEIKER – Basque Institute for Agricultural Research and Development, Basque Research and Technology Alliance (BRTA), Bizkaia Science and Technology Park 812L, 48160 Derio, Bizkaia Spain; 2 Applied Mathematics Department, Bioinformatics Unit, NEIKER – Basque Institute for Agricultural Research and Development, Basque Research and Technology Alliance (BRTA), Bizkaia Science and Technology Park 812L, 48160 Derio, Bizkaia Spain

**Keywords:** Antimicrobials, Bacteria, Pathogens, Bioinformatics, Genomic analysis, Sequencing, Microbiology

## Abstract

*Campylobacter*, a leading cause of gastroenteritis in humans, asymptomatically colonises the intestinal tract of a wide range of animals.Although antimicrobial treatment is restricted to severe cases, the increase of antimicrobial resistance (AMR) is a concern. Considering the significant contribution of ruminants as reservoirs of resistant *Campylobacter*, Illumina whole-genome sequencing was used to characterise the mechanisms of AMR in *Campylobacter jejuni* and *Campylobacter coli* recovered from beef cattle, dairy cattle, and sheep in northern Spain. Genome analysis showed extensive genetic diversity that clearly separated both species. Resistance genotypes were identified by screening assembled sequences with BLASTn and ABRicate, and additional sequence alignments were performed to search for frameshift mutations and gene modifications. A high correlation was observed between phenotypic resistance to a given antimicrobial and the presence of the corresponding known resistance genes. Detailed sequence analysis allowed us to detect the recently described mosaic *tet*(O/M/O) gene in one *C. coli*, describe possible new alleles of *bla*_OXA-61_-like genes, and decipher the genetic context of aminoglycoside resistance genes, as well as the plasmid/chromosomal location of the different AMR genes and their implication for resistance spread. Updated resistance gene databases and detailed analysis of the matched open reading frames are needed to avoid errors when using WGS-based analysis pipelines for AMR detection in the absence of phenotypic data.

## Introduction

*Campylobacter* is a leading cause of bacterial gastroenteritis throughout the industrialized world, most cases being attributed to infection with *Campylobacter jejuni* and to a lesser extent with *Campylobacter coli*^1,2^*.* Both species asymptomatically colonise the intestinal tract of a wide range of wild and domestic mammals and birds, and livestock constitute a significant source for human infection through consumption of contaminated food and water, or by contact with animals^1^. *Campylobacter* is highly prevalent in ruminants worldwide and the Basque Country (northern Spain)^3–5^, and there is increasing evidence of the significant contribution of ruminant *Campylobacter* to human campylobacteriosis^6,7^. Antimicrobial therapy is only recommended in systemic and severe *Campylobacter* infections or immunocompromised patients. However, antimicrobial resistance (AMR) is increasing globally at an alarming rate in *Campylobacter* from human and animal sources, resistance rates being usually higher in *C. coli* than in *C. jejuni*. A particular concern is its resistance to the antimicrobial agents of choice (macrolides for laboratory-confirmed cases and fluoroquinolones for cases of diarrhoea), which compromises the therapeutic efficacy^8,9^. Resistance to tetracyclines and fluoroquinolones reaches rates higher than 50% in many parts of Europe, while resistance to macrolides is lower and generally more prevalent in *C. coli*^10^. Molecular mechanisms of AMR in *Campylobacter* include point mutations, acquisition of resistance genes, and efflux systems^8^, and genetic determinants of resistance can be located on plasmids or be chromosomally encoded, which determines mechanisms of spread.

Cattle and sheep, the main livestock production systems in the Basque Country, have been shown to represent an important reservoir for resistant *Campylobacter* in the region^5,11^. The most recent study reported high rates of AMR in *Campylobacter* isolated from ruminants (cattle and sheep), with 65.1% of *C. jejuni* and 94.1% of *C. coli* isolates being phenotypically resistant to at least one of the six antimicrobial agents tested^5^. *C. jejuni* exhibited high resistance rates to fluoroquinolones (60.6%) and tetracycline (38.5%) while being mostly susceptible to aminoglycosides. In the case of *C. coli*, resistance was even higher to tetracycline (76.5%), quinolones (64.7%), and streptomycin (67.6%), and few were also resistant to erythromycin (8.8%). Molecular mechanisms associated with resistance to macrolides and fluoroquinolones were investigated using real-time PCR SNP discrimination to detect the A2075G point mutation in the 23S rRNA genes and the C257T mutation in the gyrA gene, respectively^5^. Other mechanisms were not investigated. Therefore, the aim of this study was to characterise by whole genome sequencing (WGS) the mechanisms of AMR in *C. jejuni* and *C. coli* recovered from beef cattle, dairy cattle, and sheep, focusing on the genetic diversity and population structure of resistant isolates.

## Results and discussion

### WGS quality and assembly results

Seventy *Campylobacter* (40 *C. jejuni* and 30 *C. coli*—Table S1) were selected for WGS from a collection of isolates from ruminants (beef cattle, dairy cattle, and sheep). The sequencing facility provided an average of 12.7 M ± 2.3 M of reads per sample (range = 6.7–18.6 M) corresponding to an average coverage of 1125X ± 207X (range = 593–1675X) in a 1.7 Mb genome. The median N50 of assemblies was 187 Kb (IQR = 155–228 Kb). The median number of contigs recovered per sample was 47 (IQR = 37–60). Draft genome size was estimated to vary between 1.61 and 1.87 Mb, with an overall %GC of 30.9% (Table S2). Plasmid-derived contigs were detected in 12 *C. jejuni* isolates.

### Population genomic structure

Analysis of the 70 genomes identified 36 previously described multilocus sequence types (STs) and 4 novel STs that were the result of new alleles (1 *aspA*, 1 *glnA*, 1 *glyA*, 1 *pgm* and 1 *uncA*). Thirty-seven STs were classified into 15 clonal complexes (CCs) and the remaining 3 were not assigned to any CC (they differed at two or more alleles from every other ST). Three STs included more than 5 isolates (ST-21, ST-827, ST-2097), and 27 STs included only one isolate. Irrespective of the species, considerable genetic variation was observed. However, sequence diversity was much higher among the *C. jejuni* isolates compared to the *C. coli* isolates. All but one of the 13 ST types identified among the 30 *C. coli* isolates belonged to CC-828 and the two most prevalent, ST-827 and ST-2097, accounted for 30% (n = 9) and 20% (n = 6) of the isolates, respectively. Among the 40 *C. jejuni* isolates, 27 ST types belonging to 14 CC and 2 singletons were found, the predominant clonal complex being CC-21 (n = 14, 35.0%). The most frequently observed STs in *C. jejuni* were ST-21 (n = 6, 12.5%) and ST-6532 (n = 3, 7.5%), and were the only STs that included isolates from the three animal sources; the remaining STs included one or two isolates. Whereas *C. jejuni* isolated from cattle belonged predominantly to CC-21 (n = 11) followed by CC-42 (n = 4), in *C. jejuni* isolated from sheep the most frequently found clonal complexes, CC-206 and CC-21, were represented by 4 and 3 isolates, respectively. The predominance of CC-21 among *C. jejuni* and CC-828 among *C. coli* was not unexpected, since they are known host generalist clonal complexes.

Pangenome analysis was used to compare the entire gene set of all strains and identify the core genome (present in at least 97% of the compared genomes)^12^. Out of the 3610 genes annotated among the 40 *C. jejuni* isolates compared, pangenome analyses identified 1,341 as core genes (37.1%), and a slightly higher proportion, 43.8% (1,354 of the total content of 3,090 genes) in the 30 *C. coli* isolates. Of these, 763 genes constituted the core genome of both species.

### Detection of genes and chromosomal point mutations associated with antimicrobial resistance

**Figure 1:**
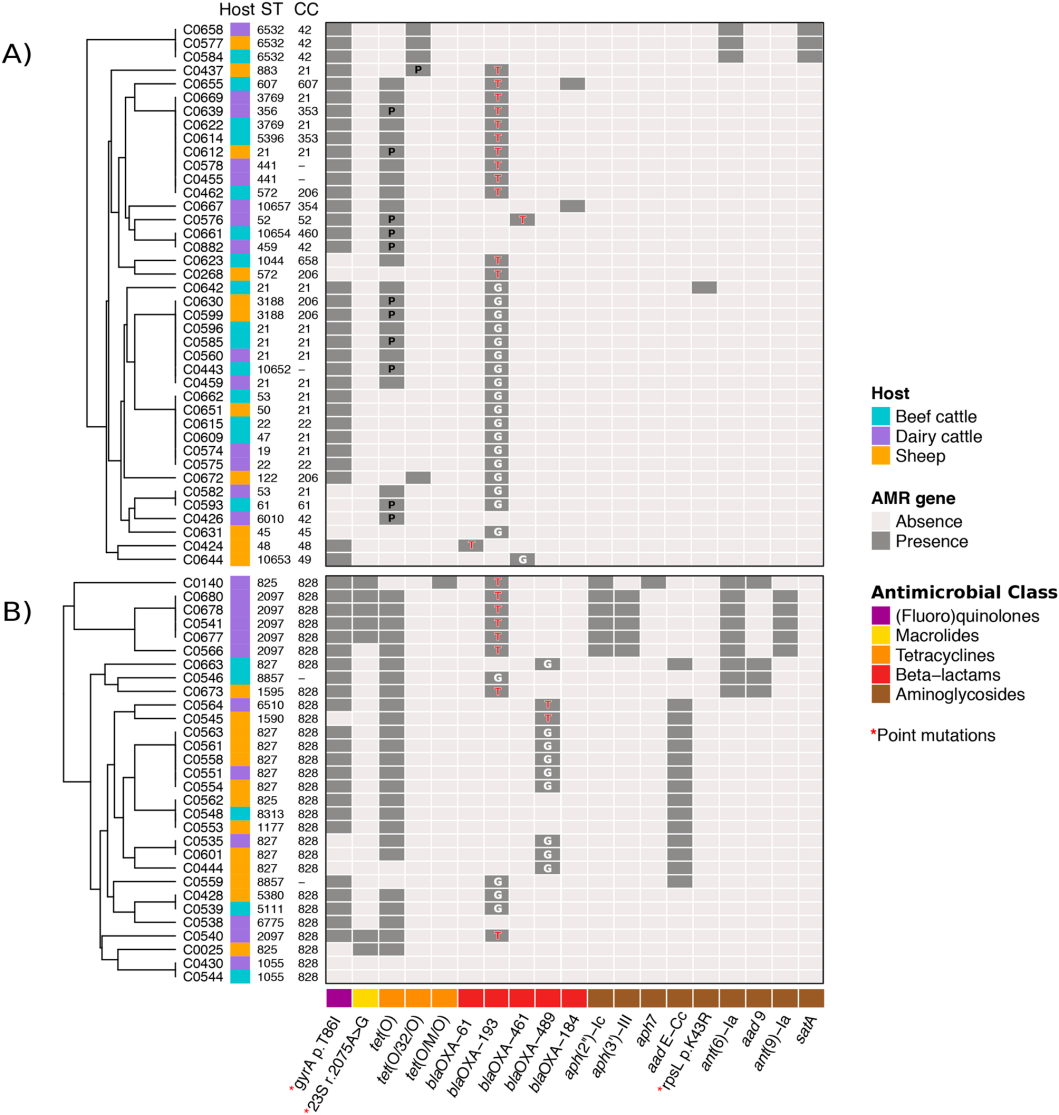
Heat map showing the distribution of antimicrobial resistance (AMR) genes detected by WGS in each isolate: (**A**) *C. jejuni*; (**B**) *C. coli*. Within each *Campylobacter* species, samples were grouped based on their antimicrobial resistance pattern according to the result of the hierarchical clustering using the average linkage method (UPGMA) on the Euclidean distance matrix. Genetic determinants of resistance are grouped according to their corresponding antimicrobial classes, which are colour coded. Assignation of each isolate to MLST profiles is indicated (ST, sequence type; CC, clonal complex). In cells corresponding to *bla*_OXA-61_-like genes, the nucleotide (G = guanine, T = thymine) at the promoter region (57 bp upstream of the start codon) is indicated (a G $\to$ T mutation is associated with high-level ampicillin resistance). Plasmid location of *tet* genes is indicated by the letter “P” in the corresponding cell.

WGS analyses identified 16 acquired AMR genes along with point mutations in another three genes that code for resistance to antimicrobials representing 5 different classes. Distribution of the genetic determinants of resistance detected by WGS in each isolate is shown in Fig. 1 along with the assignation of each isolate to MLST profiles (ST and CC).

#### Aminoglycosides

Mechanisms of aminoglycoside resistance in *Campylobacter* spp. include enzymatic drug modification^13^ and mutations at the ribosomal binding sites^14^. Genes coding for aminoglycoside-modifying enzymes of two distinct families were found in this study, aminoglycoside phosphotransferases (APH) and aminoglycoside nucleotidyltransferases (ANT), along with the sporadic detection of a point mutation in *rpsL* gene. APHs in *Campylobacter* are mainly encoded by *aph(3′)-III*, which confers resistance to amikacin, and *aph(2′')-Ic* gene, which confers resistance to gentamicin. In this study, *aph(2′')-Ic* gene was found in the 6 gentamicin-resistant *C. coli* (MIC > 16 mg/L), along with *aph(3′)-III* in 5 of them. This *aph(2′')-Ic* gene presented a 3 nucleotide (nt) gap in all 6 isolates compared to the reference sequences in GenBank, which resulted in the loss of Tyrosine (Y) at aa position 8. They were all resistant to gentamicin.

ANTs in *Campylobacter* include ANT(6) and ANT(9), which confer resistance to streptomycin and spectinomycin, respectively, and can be encoded by different genes^13,15^. Here, streptomycin resistance was coded by *ant(6)-Ia* (3 *C. jejuni* and 9 *C. coli*) and *aadE*-Cc (12 *C. coli*). Besides, a point mutation (K43R) in *rpsL* conferred resistance to STR in one *C. jejuni*. The gene *aad9*, also associated with aminoglycoside resistance was found in four *C. coli* isolates. Sequences that only shared 75.5% identity with *aadE*-Cc (CP013733) but were 100% identical to a gene in *C. coli* strain ZV1124 (CP017875) that encodes an aminoglycoside 6-adenylyltransferase (*C. coli* WP002814927) were identified in three other *C. coli*; one isolate phenotypically resistant to streptomycin (C0601, MIC = 8 mg/L) and another two streptomycin-susceptible *C. coli* isolates (C0444, MIC = 2 mg/L & C0558, MIC = 4 mg/L). Another three *C. jejuni* phenotypically susceptible to aminoglycosides carried a gene that was 99.4% identical to a streptomycin aminoglycoside 6-adenyltransferase gene (LR134496.1:1,509,775–1,510,653 *Campylobacter jejuni* strain NCTC13266) but turned out to be non-functional due to a frameshift mutation at nt position 394 that caused truncation of the ORF. No genetic determinant of resistance (GDR) was found in one *C. coli* (C0430) that showed a MIC = 8 mg/L for streptomycin, just one dilution step above the ECOFF for resistance.

**Figure 2:**
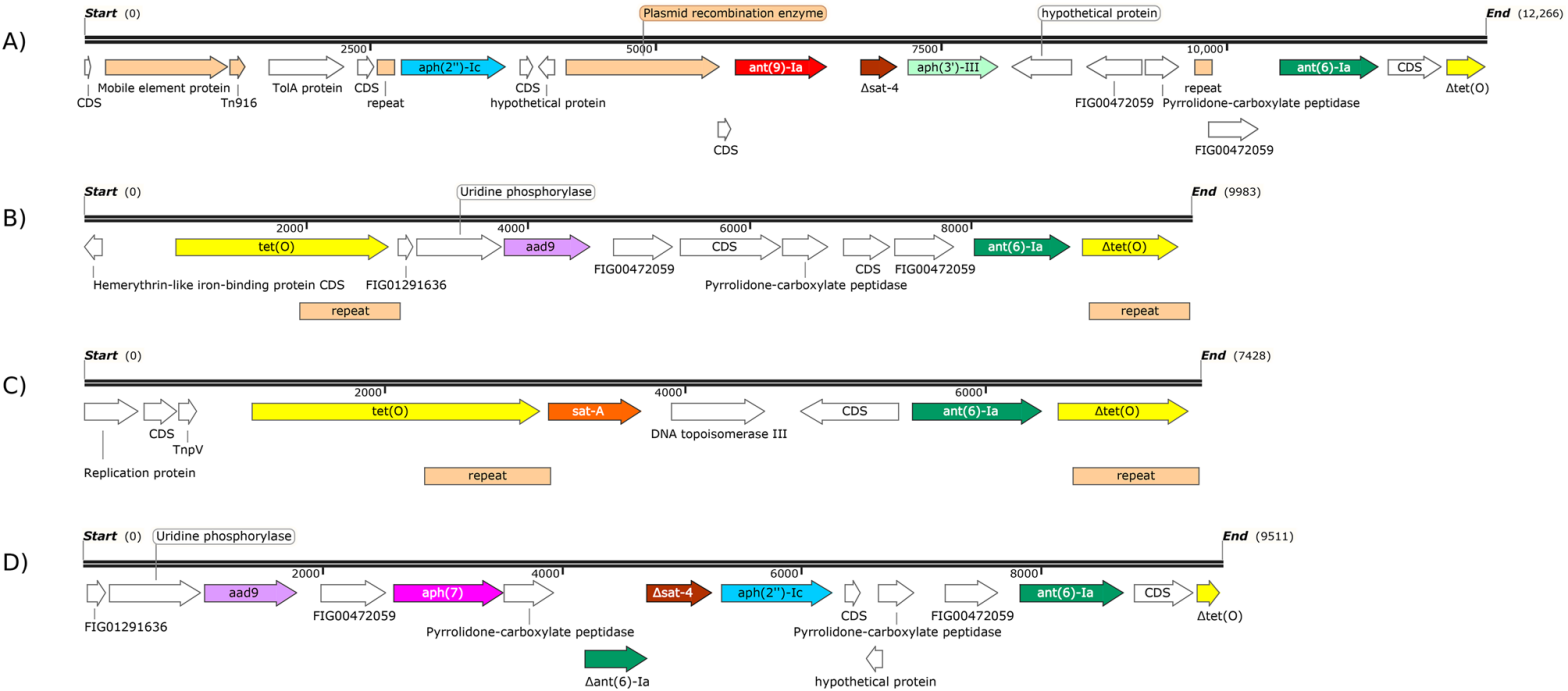
Aminoglycoside resistance clusters identified in nine *C. coli* and three *C. jejuni* isolates. Each coding sequence (CDS) is represented by an arrow with the direction indicating the translational direction. $\Delta$, truncated gene.

The aminoglycoside resistance genes in *Campylobacter* have been detected on multidrug-resistant plasmids, integrons, transposons and chromosomal aminoglycoside resistance islands^16^. Here, aminoglycoside resistance clusters were identified in nine *C. coli* and three *C. jejuni* isolates, located in contigs of chromosomal origin as determined by PlasFlow. Based on the genes present and their relative locations they were classified into four types. The most abundant cluster (5 isolates) included six AMR genes, *i.e.*, *aph(2′')-Ic*, *ant(9)-Ia*, a truncated $\Delta$*sat-4*, *aph(3′)-III*, *ant(6)-Ia* and a truncated $\Delta$*tet*(O). Several mobile genetic elements were found upstream the *aph(2′')-Ic* gene, and included a mobile element protein that shared 72% identity with an IS30 family transposase from *Megasphaera elsdenii*, a Tn916 100% identical to a conjugal transfer protein from *C. coli*, a plasmid recombination enzyme found in several Gram positives, and two 151 bp interspersed direct repeats (Fig. 2A). This cluster type was found in 5 *C. coli* isolated from dairy cattle in 4 different farms located in the same county, they were all phenotypically resistant to AMP-CIP-GEN-NAL-STR-TET (four of them also to ERY), belonged to the same MLST type (CC-828, ST-2097, same cgMLST). The second type of cluster was comprised of *tet*(O), *aad9*, *ant(6)-Ia*, and a truncated $\Delta$*tet*(O) gene. Two 908 bp direct repeats located downstream each *tet*(O) sequence flanked the aminoglycoside cluster (Fig. 2B). It was found in two *C. coli* isolated from beef cattle (one without *tet*(O), probably because of the shorter size of the contig) and another *C. coli* from sheep in three different farms located in the same county. All three were phenotypically resistant to CIP-NAL-STR-TET, all carried *bla*_OXA_ but only two were AMP resistant, and they belonged to different ST types. The third cluster consisted of *tet*(O), *sat-A*, *ant(6)-Ia*, and $\Delta$*tet*(O) genes. Upstream the *tet*(O) gene, a *C. jejuni* plasmid replication protein and a transposon-encoded protein TnpV were found. Also, two 838 bp direct repeats were located downstream of each *tet*(O) sequence (Fig. 2C). This cluster was present in three *C. jejuni* isolated from beef cattle, dairy cattle and sheep in three farms from different counties that had the resistance profile CIP-NAL-STR-TET and the MLST type ST-6532 (CC-42). Finally, one *C. coli* isolated from dairy cattle (ST-825, CC-828) and resistant to AMP-CIP-GEN-NAL-STR-TET-ERY, carried the resistance cluster in a shorter contig (9,512 bp) that harboured *aad9*, *aph7*, a truncated $\Delta$*ant(6)-Ia*, a truncated $\Delta$*sat*-4, *aph(2′')-Ic*, *ant(6)-Ia*, and a truncated $\Delta$*tet*(O) (Fig. 2D). No mobile genetic elements or repeat sequences were found, but several hypothetical proteins found shared high similarity with several Gram-positive microorganisms.

These gene cassettes might have undergone spread by horizontal gene transfer, as suggested by the presence of several mobile genetic elements. Besides, the similarity of genes encoding aminoglycoside-modifying enzymes and several other flanking genes with genes from Gram-positive bacteria suggests that they might have originated from Gram-positive bacteria^16^. On the other hand, the high genetic similarity of isolates harbouring cluster 1, and the fact that they all originated from the same area suggested that these isolates might have originated from a single clone by clonal expansion. Also noteworthy is that not all of the aminoglycoside resistance genes found in the cassettes encoded functional ORFs; some were shown to be truncated and not functional. Finally, *aadE*-Cc, the most common streptomycin resistance coding gene in *C. coli*, was never located within these aminoglycoside resistance gene clusters.

#### Tetracyclines

Specific tetracycline resistance in *Campylobacter* is associated with genes encoding ribosomal protection proteins (RPPs)^17^. Here, fully functional tetracycline resistance encoding genes were found in all 56 phenotypically resistant isolates, with *tet*(O) gene being the most prevalent (n = 50), along with two mosaic genes, *tet*(O/32/O) in 5 *C. jejuni* and *tet*(O/M/O) in 1 *C. coli*. Until recently, the only RPP mosaic gene described in *Campylobacter* was *tet*(O/32/O). However, the *tet*(O/M/O) gene, where the 777–1126 bp internal fragment of *tet*(O) is replaced by the homologous sequence from *tet*(M), has just been recently described^18,19^. Although it is present in several *C. jejuni* and *C. coli* genomes in the databases, it is not commonly reported in studies dealing with molecular detection of AMR probably due to their absence from AMR databases. Here, partial matching using automated annotation pipelines prompted us to further analyse the sequence. Compared to other sequences in GenBank, our sequence had 3 nt mutations compared to other *tet*(O/M/O) gene sequences that resulted in 2 aa changes, E38K and C595N. Given the wide distribution of *tet*(M) in different bacteria and the previous reports of self-recombination^17^, the true prevalence of this type of mosaic gene may be higher than reported.

In *Campylobacter*, Tet coding genes can be located both in the chromosome and in plasmids. Here, they were chromosomally encoded in 41 isolates and located in plasmids in 12 *C. jejuni* isolates (Fig. 1); in another 3 isolates, they were found in contigs that were too small to reliably predict the location. Four of the 12 *C. jejuni* plasmids were fully sequenced and turned out to be nearly identical among themselves and highly similar to *C. coli* strain FB1 plasmid pFB1TET (Acc. Nr. CP011017). The four circular plasmids were about 48.8 kb in size (Fig. S1). The remaining 8 plasmid-derived contigs were shorter in size and represented partial plasmids. They all shared the highest similarity with pTet plasmids (Type 1), and included *tet*(O) and several Type IV secretion system (T4SS) genes described to form the core genome of pTet plasmids in *Campylobacter*^20^. The tetracycline resistance encoding gene found in the pTet plasmids was *tet*(O) in 11 isolates and *tet*(O/32/O) in one. In addition, other elements present in the complete plasmids included a site-specific recombinase (resolvase family), IncQ plasmid conjugative transfer proteins, phage Rha protein, virulence-associated protein 2 (VapD), and a cag pathogenicity island protein (cag12). No other AMR-coding genes were found in these plasmids. pTet plasmids have been reported to be the most prevalent plasmid type in *C. jejuni* and *C. coli* clinical isolates in Germany^21^ and isolates from retail meat sources in the USA^20^. Here, they were equally distributed among dairy cattle, beef cattle and sheep (4 isolates each), and included *C. jejuni* isolates from different lineages (10 different ST and 8 CC) (Fig. 1).

#### β-lactams

Fifty-seven isolates carried a gene coding for oxacillinases of the OXA-61-like family (n = 55), OXA-184-like family (n = 1), or both (n = 1). One *C. jejuni* isolate carried the *bla*_OXA-184_ gene, another *C. jejuni* carried both *bla*_OXA-184_ and *bla*_OXA-193_, and the remaining 55 isolates carried different *bla*_OXA-61_-like gene alleles, *i.e.*, *bla*_OXA-193_ (29 *C. jejuni* and 12 *C. coli*), *bla*_OXA-489_ (11 *C. coli*), *bla*_OXA-461_ (2 *C. jejuni*) and *bla*_OXA-61_ (1 *C. jejuni*). β-lactams are not recommended for treating campylobacteriosis, and therefore, in 2007 EFSA considered them as optional for monitoring at the EU level^22^. Nowadays, most of the panels used for routine AMR monitoring for *Campylobacter* spp. do not include this class of antimicrobials. In a previous study where β-lactams were tested in the region^11^, microbiological resistance to ampicillin and amoxicillin was 7.1% in *C. jejuni* isolates from beef cattle (1/14), 35.7% (5/14) in dairy cattle, and 20% (5/25) in sheep. Considering the widespread distribution of *bla*_OXA_ found in the study herein, microbiological resistance to AMP was determined by E-test to complement the microdilution plate panel that did not include any β-lactam. Also, *bla*_OXA_ sequences were subjected to a more detailed analysis that included both the promoter and the coding region.

Based on E-test results, 25 isolates (11 *C. coli* and 14 *C. jejuni*) were phenotypically resistant to AMP. Analysis of the *bla*_OXA-61_-like promoter region identified the presence of a guanine (G) $\to$ thymine (T) point mutation (57 bp upstream of the start codon) associated with high-level ampicillin resistance in 24 isolates. The G $\to$ T transversion has been described to restore the TATA box (from GAAAAT to TAAAAT) making it fully functional thus increasing oxacillinase production and consequently causing high-level ampicillin resistance^23^. In general, the presence of a G at the *bla*_OXA-61_-like promoter region was associated with a susceptible phenotype, whereas a T mutation caused resistance. The exceptions were a *bla*_OXA-489_-carrying *C. coli* isolate (C0663) that was AMP-resistant (E-test MIC = 16 mg/L) but did not have the mutation at the promoter, and the *bla*_OXA-61_-carrying *C. jejuni* isolate (C0424) that had the mutation but was AMP-susceptible. The only difference found in *C. coli* C0663 was a unique mutation (C $\to$ T) 77 bp upstream of the start codon of *bla*_OXA-489_ of unknown effect. Analysis of the *bla*_OXA-61_ gene in *C. jejuni* C0424 produced more intriguing results. It had an insertion (T at nt position 724) which changed the reading frame and resulted in an in-frame TGA codon that could be translated into selenocysteine (Sec) and encode a 253 aa protein or be decoded as a termination signal and produce an even shorter protein of 247 aa. In both cases, the resulting protein would share amino-acid similarity for the first 241 amino acids, with the former having FRKIFR at the C-terminal end and the latter ending FRKIFRUTCKKS. The secondary structure or the integrity of the transpeptidase structural domain would not be affected by either of these shortens in length. The 21st amino acid, Sec, has already been identified in the FdhA subunit of *C. jejuni* formate dehydrogenase (FDH) enzyme^24^, and in several other bacteria, archaea, and eukaryotes^25^. Alternatively, if decoded as a termination signal, it would result in premature termination of protein synthesis producing in a 247 aa protein. While the bla_OXA-193_ gene encodes a 257 aa oxacillinase, fully functional shorter OXA proteins (*eg*. *bla*_OXA-460/461_ encoding 253 amino acids) have already been described^26^ and were also found here. The shorter length of genetic determinants has been attributed to extreme environmental conditions and ecological adaptations^27^. Although the transpeptidase structural domain would not be altered, this isolate was susceptible to ampicillin despite harbouring a T in the promoter region. Further studies are needed to identify the mechanisms affecting gene expression in this isolate.

Other minor mutations found when analysing the *bla*_OXA_ sequences in detail included one or two aa substitution in the *bla*_OXA-193_ gene of three isolates (A43V, 1 *C. jejuni*; I20V and M98I, 2 *C. jejuni*), all harbouring the wild-type G at the promoter, and one aa substitution (L48S) in the *bla*_OXA-461_ gene of another *C. jejuni*. Several other synonymous substitutions were also found in 44 isolates, the most widespread being a G to A transition at nt position 24.

#### (Fluoro)quinolones

Resistance to quinolones was in all cases (n = 57) coded by a chromosomal point mutation in the *gyrA* gene (C257T; T86I), all isolates carrying this mutation being resistant to both CIP and NAL. The only isolate that was phenotypically resistant to only one of the two quinolones, *i.e.*, one *C. jejuni* (C0268) susceptible to NAL (MIC = 16 mg/L) but resistant to CIP (MIC = 1 mg/L), did not carry any point mutation in the *gyrA* gene. Although point mutations at multiple positions of the DNA gyrase A (GyrA) region can cause resistance towards fluoroquinolones in *Campylobacter*^8^, the Thr86Ile GyrA mutation has been reported as the most prevalent mechanism in *Campylobacter* from animal and human sources^8,28–30^. This is in agreement with the results presented here and with previous studies where this mutation was detected by SNP-PCR in fluoroquinolone-resistant *C. jejuni* isolated from ruminants and poultry in the region^5,11^.

#### Macrolides

All 7 erythromycin-resistant *C. coli* isolates (MIC > 128 mg/L) carried a point mutation (A2075G) in the 23S rRNA gene, which was not present in susceptible isolates (all 40 *C. jejuni* and 23 *C. coli*). This is the most prevalent genetic determinant conferring resistance to erythromycin^8,28,29^. Carriage of the *ermB* gene, usually present on MDRI or plasmids bearing other resistance genes, has been associated with a high-level of erythromycin resistance (MIC > 128 mg/L)^31^. Here, 128 mg/L was the highest concentration tested, and none of the isolates harboured mutations at position 2074 of the 23S rRNA gene or carried the *ermB* (erythromycin ribosome methylation) gene. Similarly, the point mutations G86A in the transcriptional regulator CmeR of the multidrug efflux pump CmeABC system, associated with an increased level of resistance towards macrolides, fluoroquinolones, and tetracycline^32^, was not found in any of the isolates.

### Comparison between phenotype (MIC) and genotype (WGS) data: correlation of susceptibility phenotypes and genotypes

In agreement with other WGS-based studies^28,30^, there was an overall very good concordance between susceptibility phenotypes and genotypes as shown in Table 1 (Results of the validity tests: sensitivity, specificity, average PPV and NPV, Cohen’s kappa score). The presence of known AMR coding genes and/or chromosomal point mutations accurately predicted phenotypic resistance to GEN, TET, NAL, and ERY, while minor discrepancies were found for CIP, STR, and AMP. Thus, no point mutation in the *gyrA* gene was identified in the genome of one *C. jejuni* isolate (C0268) that was phenotypically resistant to CIP (MIC = 1 mg/L) and susceptible to NAL (MIC = 16 mg/L). Most CIP-resistant *Campylobacter* are also resistant to NAL, this isolate being the only exception in this study. The MIC for ciprofloxacin was just a single two-fold dilution above the ECOFF, and therefore, within the widely accepted margin of error of the method. The presence of the aminoglycoside 6-adenylyltransferase gene (WP002814927) in two *C. coli* isolates did not result in phenotypic resistance to streptomycin (C0444, MIC = 2 mg/L & C0558, MIC = 4 mg/L), whereas no GDR was found in one *C. coli* phenotypically resistant to streptomycin (C0430, MIC = 8 mg/L). The presence of a *bla*_OXA_ gene along with the T mutation at the promoter provided a good WGS-based prediction of resistance; however, two discrepancies were still found. Further sequences analyses highlighted small sequence changes that would need further investigation to ascertain if they represented fully functional genes.

**Table 1:** Concordance tests between phenotypic antimicrobial susceptibility testing and WGS-based predicted antimicrobial resistance.

Antimicrobial agent	Sensitivity		Specificity		Predictive value +		Predictive value −		Cohen's kappa coefficient			
	%	95% CI	%	95% CI	%	95% CI	%	95% CI	κ	95% CI	*p* value	Interpretation
Ampicillin (AMP)^a^	96.0	80.5 to 99.3	96.0	88.4 to 99.6	96.0	80.5 to 99.3	97.8	88.4 to 99.6	0.94	0.85 to 1.00	< 0.001	Very Good
Ciprofloxacin (CIP)	98.2	90.7 to 99.7	100.0	75.8 to 100	100.0	93.6 to 100	92.3	66.7 to 98.6	0.95	0.86 to 1.00	< 0.001	Very Good
Nalidixic acid (NAL)	100.0	93.6 to 100	100.0	77.2 to 100	100.0	93.6 to 100	100.0	77.2 to 100	1.00	1.00 to 1.00	< 0.001	Very Good
Erythromycin (ERY)	100.0	64.6 to 100	100.0	94.2 to 100	100.0	64.6 to 100	100.0	94.2 to 100	1.00	1.00 to 1.00	< 0.001	Very Good
Gentamicin (GEN)	100.0	61.0 to 100	100.0	94.3 to 100	100.0	61.0 to 100	100.0	94.3 to 100	1.00	1.00 to 1.00	< 0.001	Very Good
Streptomycin (STR)	96.2	81.1 to 99.3	95.3	84.5 to 98.7	92.6	76.6 to 97.9	97.6	87.7 to 99.6	0.91	0.81 to 1.00	< 0.001	Very Good
Tetracycline (TET)	100.0	93.6 to 100	100.0	77.2 to 100	100.0	93.6 to 100	100.0	77.2 to 100	1.00	1.00 to 1.00	< 0.001	Very Good

^a^Presence of *bla*_OXA-61_-like gene with T at the promoter region (57 bp upstream of the start codon) and/or presence of *bla*_OXA-184_ gene.

### Association of MLST sequence types with AMR

**Figure 3:**
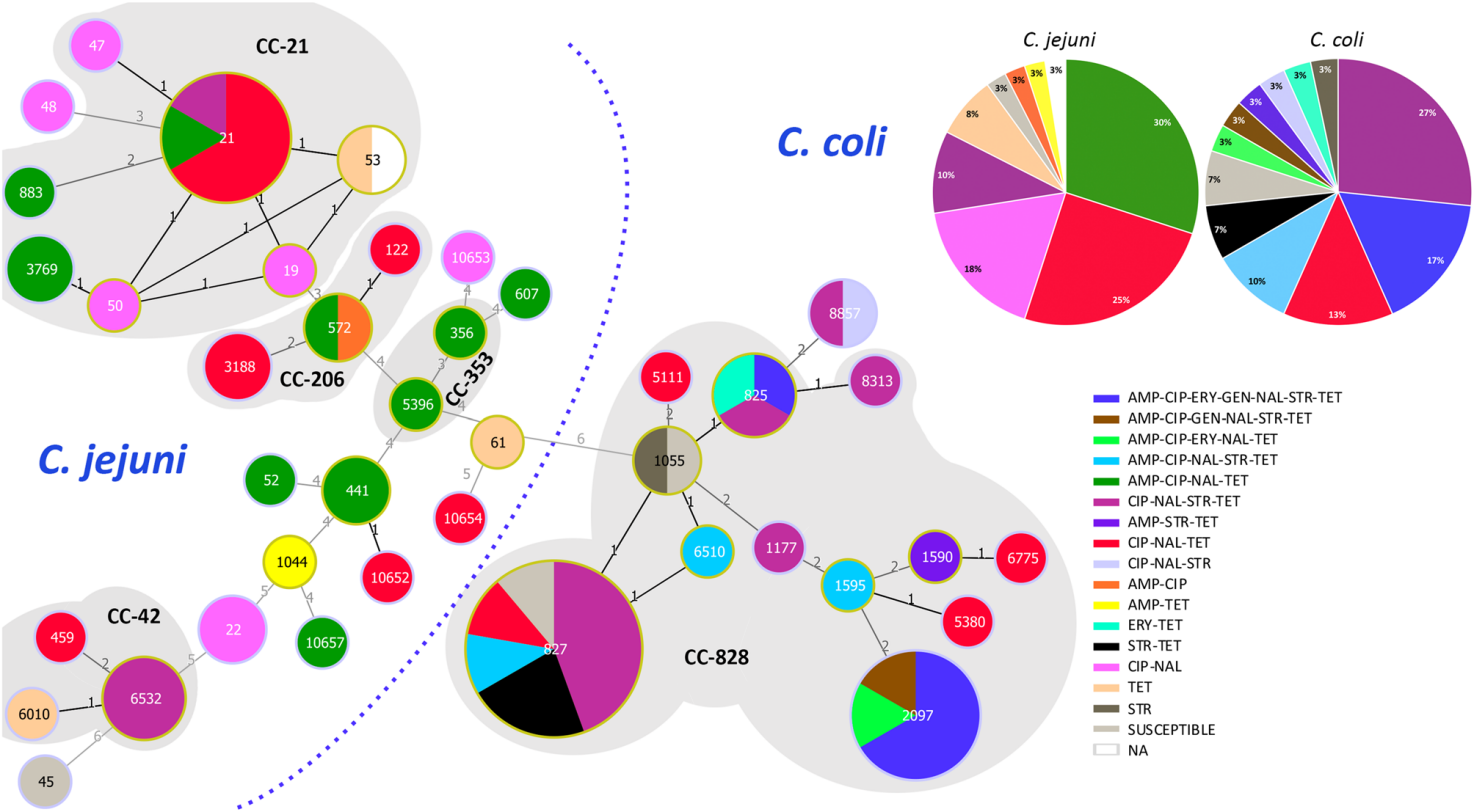
Minimum spanning tree of *C. jejuni* and *C. coli* isolates constructed from MLST profiles showing the distribution of the different phenotypic resistance profiles. Each node represents an ST type and the size of the node correlates to the number of isolates. Clonal complexes (CC) represented by more than one ST type are highlighted in grey shadow and the corresponding CC is indicated. Phenotypic antimicrobial resistance profiles shown as percentages are given for *C. jejuni* and *C. coli* in separate pie charts.

The relatedness of different sequence types (ST, CC) and their associations with antimicrobial resistance patterns is depicted in Figs. 1 and 3. Although the limited number of isolates and the high genetic diversity observed hampers the possibility to establish statistically supported associations, some patterns were commonly found in association with the same sequence types (ST, CC). For example, in *C. coli*, ST-2097 isolates showed a highly similar AMR gene profile. ST-2097 is not a very frequently encountered type according to records in the *Campylobacter* PubMLST database, which only includes 8 isolates; 4 from humans, 1 from an unknown source, and, interestingly, 3 from chicken isolated in Spain (https://pubmlst.org/bigsdb?db=pubmlst_campylobacter_isolates; last searched 14 December 2020). In *C. jejuni*, ST-6532 (CC-42) was associated with the same AMR profile but the remaining CC-42 isolates (ST-459 and ST-6010) lacked the aminoglycoside cluster (both) and the mutation T86I at *gyrA* (ST-6010). Although CC-42 is considered a ruminant-associated clonal complex, in the *Campylobacter* PubMLST database ST-6532 is represented by 9 human isolates and ST-6010 by one environmental isolate from a farm in the Basque Country. On the other hand, 9 of the 11 *C. coli* isolates that harboured the *bla*_OXA-489_ gene belonged to ST-827 and none carried the mutation at the promoter and were therefore susceptible to AMP, whereas the remaining 2 isolates (ST-1590 and ST-6510) carried the mutation and were resistant.

## Conclusion

WGS was used to characterise and predict AMR in a collection of *C. jejuni* and *C. coli* with different resistance phenotypes isolated from a representative sample of beef cattle, dairy cattle, and sheep farms in the Basque Country. Genome analysis showed the extensive genetic diversity of the selected collection of isolates. When used to predict antimicrobial resistance, comparison of the resistance genotypes to their respective phenotypic resistance profiles showed very high accuracy, with a total agreement between phenotypic and genotypic results for GEN, TET, NAL, and ERY (Cohen’s kappa κ = 1), and κ above 0.9 for CIP, STR, and AMP. However, frameshift mutations leading to non-functional gene variants and mosaic genes remain a problem when using automated annotation pipelines. Updated databases and detailed analysis of the matched open reading frames are needed to avoid such errors when using WGS-based analysis pipelines for AMR detection in the absence of phenotypic data. Such detailed sequence analysis allowed us to detect the recently described mosaic *tet*(O/M/O) gene in one *C. coli* isolated from dairy cattle, and describe possible new alleles of *bla*_OXA-61_-like genes. The AMR genes identified in the present study were mostly located on the bacterial chromosome, but pTet plasmids that harboured the *tet*(O) gene and several T4SS were also identified in 12 *C. jejuni* isolates from different sources and ST types, suggesting intra-species dissemination of these type of plasmids. The analysis of the genetic context of aminoglycoside resistance genes showed different gene organisations and lineage distributions, in some instances suggesting mobilisation of gene clusters by horizontal transfer, and also proliferation by clonal expansion after the acquisition of AMR genes in a single ancestral event in other cases.

## Methods

### Bacterial isolates: selection and available data

Seventy *Campylobacter* (40 *C. jejuni* and 30 *C. coli*) were selected from a collection of isolates from ruminants (beef cattle, dairy cattle, and sheep). Most of them (66) were isolated during a cross-sectional survey carried out between February 2014 and June 2016 to estimate the prevalence of *C. jejuni* and *C. coli* in ruminants in the Basque Country (Northern Spain)^5^, another three were isolated 10 years earlier (2004–2005) as part of a similar study^33^, and one in 2019. Phenotypic antimicrobial resistance data (minimum inhibitory concentration, MIC) against six antimicrobial agents (gentamicin, streptomycin, tetracycline, ciprofloxacin, nalidixic acid, and erythromycin) were available for all isolates and resistance rates to the abovementioned antimicrobials in *C. jejuni* and *C. coli* isolated from the different hosts were reported elsewhere^5,33^. Isolates were selected among those that exhibited microbiological resistance to at least one of the six antimicrobial agents tested (only one *C. jejuni* and two *C. coli* susceptible isolates were included) to represent the different resistance phenotypes. For sample selection, available isolates were stratified to ensure a similar representation of the different farms and locations, and the different hosts (beef cattle, dairy cattle, and sheep). Twenty-two isolates (4 *C. jejuni* and 18 *C. coli*) were multidrug-resistant (defined as resistance to ≥ 3 classes of antimicrobial drugs) based on the analysis to the abovementioned antimicrobials. In all cases, isolates had been recovered from rectal faeces collected from apparently healthy animals in 68 farms (20 beef cattle, 24 dairy cattle, and 24 sheep) located throughout the Basque Country. Isolate metadata and phenotypic antimicrobial resistance data are described in Table S1.

### Antimicrobial susceptibility testing

Minimum inhibitory concentrations (MICs) against gentamicin (GEN), streptomycin (STR), tetracycline (TET), ciprofloxacin (CIP), nalidixic acid (NA), and erythromycin (ERY) were previously determined using EUCAMP2 Sensititre MIC susceptibility plates (Thermo Fisher Scientific, Waltham, MA)^5^ following recommendations by the Commission Decision 2013/652/EU. Results were interpreted using epidemiological cut-off values (ECOFF) as developed by the European Committee for Antimicrobial Susceptibility Testing (EUCAST; http://www.eucast.org). ECOFFs were used to define microbiological resistance to the antimicrobial in question, that is, to discriminate those microorganisms with and without acquired resistance mechanisms (non-wild type and wild type, respectively), as follows: AMP > 8 mg/L; CIP > 0.5 mg/L, ERY > 8 mg/L (*C. coli*) and > 4 mg/L (*C. jejuni*); GEN > 2 mg/L; NAL > 16 mg/L; STR > 4 mg/L; TET > 2 mg/L (*C. coli*) and > 1 mg/L (*C. jejuni*).

Resistance to ampicillin (AMP) was determined using E-test strips (bioMérieux) (concentration range: 0.016–256 mg/L) following the manufacturer`s instructions. Several well-isolated colonies from overnight growth on Columbia agar were suspended in cation-adjusted Mueller Hinton broth with TES (ThermoFisher Scientific) to a turbidity of 0.5 McFarland. The suspension was swabbed onto Mueller Hinton agar with 5% horse blood + 20 mg/L ß-NAD (MHF, bioMérieux), and one E-test strip was placed onto the inoculated agar. Plates were incubated at 37 °C under microaerophilic conditions for 48 h. MIC values were read at the pointed end where the inhibition ellipse intersects the MIC scale of the strip. EUCAST ECOFF (MIC > 8 mg/L) was used for the interpretation.

### Whole-genome sequencing (WGS) and genome assembly

All *Campylobacter* strains, stored in liquid nitrogen since isolation, were subcultured on Columbia agar supplemented with 5% of sheep blood (bioMerieux) and incubated for 48–72 h under microaerobic conditions (5% O_2_, 10% CO_2_, and 85% N_2_) at 42 °C. DNA was extracted from single-colony cultures (NZY Microbial gDNA Isolation kit, NZYtech) and submitted to a commercial facility where libraries were prepared based on the NEBNext Ultra™ II FS DNA Library Prep Kit (Illumina) and sequenced using Illumina NovaSeq6000. Quality of raw reads (2 × 150 bp paired-end) was assessed using FastQC v.0.11.9^34^, and Trimmomatic v.0.38^35^ was used to trim Illumina adapters. Low-quality reads (reads with a quality score < 25 over a sliding window size of 15 bp, and reads with a sequence length < 125 bp) were filtered out using PRINSEQ v.0.20.4^36^, and de novo assembled using SPAdes v.3.13.0^37^. The quality of the assemblies was assessed with QUAST v.5.0.2^38^, discarding contigs below 200 bp with PRINSEQ v.0.20.4^36^.

### WGS analysis: antimicrobial resistance (AMR) determinants and sequence typing (ST)

BLASTn v.2.9.1 + ^39^ and ABRicate v.1.0.0 (https://github.com/tseemann/abricate) were used to screen for acquired antimicrobial resistance genes against ResFinder^40^, NCBI^41^, ARG-ANNOT^42^, CARD^43^, and MEGARes^44^ databases (last updated on November 16, 2020). Chromosomal point mutations associated with antimicrobial resistance were identified with PointFinder v.3.1.0^45^ (last updated on May 5, 2020). AMR gene sequences showing gaps/insertions, less than 90% identity and/or 60% coverage were further examined to identify the possible effect of the mutations on translated proteins or the presence of truncated genes in small contigs. PointFinder was not able to detect point mutations in the *gyrA* and *cmeR* genes among *C. coli* isolates, so these regions were extracted with Geneious (Geneious version 2020.1 created by Biomatters. Available from https://www.geneious.com) and aligned using MEGAX v.10.1.8^46^ to manually identify the mutations at codon positions 70, 85, 86, 90 and 104 for the *gyr*A gene and at codon positions 86 for the *cmeR* gene. The same procedure was followed to identify the presence of a G $\to$ T point mutation in the *bla*_OXA-61_-like promoter region in both *C. jejuni* and *C. coli* by exporting and comparing 100 nt flanking the *bla*_OXA-61_-like coding region to capture the mentioned mutation at position 57 upstream of the starting codon. Multilocus sequence types (MLST) were queried against the *Campylobacter* MLST database pubMLST^47^ using mlst, and core-genome MLST types (cgST) were assigned using cgMLSTFinder v.1.1 (https://bitbucket.org/genomicepidemiology/cgmlstfinder/^48^) following the pubMLST *Campylobacter jejuni/coli* cgMLST scheme^47^ (last updated on May 22, 2020). Novel alleles were submitted to the *Campylobacter* MLST database for allele and sequence type (ST) assignations. Minimum Spanning Tree was constructed by the goeBURST algorithm using the Phyloviz v2.0 software^49^ to visualize the relationships between the STs and the distribution of AMR phenotypic profiles.

RFPlasmid^50^ and PlasFlow^51^ were used to predict plasmid- and chromosome-derived contigs. Both tools rely on artificial intelligence models (Random Forest and Neural Networks, respectively) to accurately predict sequences' origin (whether if they come from a plasmid or a chromosome) or even characterise them phylogenetically (based on the bacterial sequences used for the model’s training). Plasmid-derived contigs and contigs that carried several AMR genes were annotated using the Department of Energy Systems Biology Knowledgebase (KBase) annotation app (available at https://www.kbase.us/) that uses components from the RASTtk v.1.073^52–54^. The resulting gbff files were used to reconstruct the genetic context using SnapGene Viewer® software (from GSL Biotech; available at snapgene.com) (aminoglycoside clusters). The BLAST Ring Image Generator (BRIG) software^55^ was used for comparison analysis of plasmids and image generation of Fig. S1. Based on the annotation of the draft genomes using Prokka v.1.14.6^56^, pangenomes for *C. coli* and *C. jejuni* were inferred using Roary v.3.13.0^57^ which was run adjusting -e, -n, and -cd 97 parameters. Those genes present in at least 97% of the isolates were considered core genome.

A heatmap illustrating the presence of the different AMR determinants in each sample was created, along with a dendrogram illustrating the similarity among isolates based on their AMR pattern. The hierarchical clustering analysis for the dendrogram was performed with the unweighted pair-group method with arithmetic mean (UPGMA) based on the Euclidean distance matrix, using the function hclust (v.3.6.1) of the R statistical package v.3.6.3^58^.

### Statistical analysis

Phenotypic (broth microdilution AST-based) and genotypic (WGS-based) susceptibility results were compared. Resistant WGS genotypes were defined by the presence of one or more resistance genes and/or point mutation for each antimicrobial tested in the AST. The sensitivity, specificity, and positive (PPV) and negative (NPV) predictive values for the genotypic prediction were calculated for each antimicrobial tested for their corresponding phenotypic AST reference. Inter-rater agreement analyses were performed for each antimicrobial using Cohen's kappa (κ) method. Interpretation of Kappa values to assess the strength of agreement between techniques was based on the one proposed by Altman^59^, which is as follows: κ ≤ 0.20 = poor, 0.21–0.40 = fair, 0.41–0.60 = moderate, 0.61–0.80 = good, and 0.81–1.00 = very good. Analyses were conducted using statistical software Stata/IC version 13.1 (StataCorp LP, College Station, TX, USA).

## Supplementary Information

Supplementary Information 1.

## Data Availability

Sequencing data of the 70 genomes analysed in this study have been deposited at the NCBI Sequence Read Archive (SRA) database under accession numbers SRR13362733 to SRR13362802, associated with the BioProject accession number PRJNA689687 (BioSamples SAMN17214743 to SAMN17214812).
