# Supplementary material for: Whole genome-based characterisation of antimicrobial resistance and genetic diversity in Campylobacter jejuni and Campylobacter coli from ruminants
Source: Sci Rep. 2021 Apr 26;11:8998. doi: 10.1038/s41598-021-88318-0 (PMC8076188; doi:10.1038/s41598-021-88318-0)
Supplement: Supplementary file 1 — Supplementary Information 1. [file 41598_2021_88318_MOESM1_ESM.pdf]

Whole genome-based characterisation of antimicrobial resistance and genetic diversity in *Campylobacter jejuni* and *Campylobacter coli* from ruminants

Medelin Ocejo[1], Beatriz Oporto[1], José Luis Lavín[2] and Ana Hurtado[1,*]

[1] Animal Health Department, NEIKER – Basque Institute for Agricultural Research and Development, Basque Research and Technology Alliance (BRTA), Bizkaia Science and Technology Park 812L, 48160 Derio, Bizkaia, Spain.

[2] Applied Mathematics Department, Bioinformatics Unit, NEIKER – Basque Institute for Agricultural Research and Development, Basque Research and Technology Alliance (BRTA), Bizkaia Science and Technology Park 812L, 48160 Derio, Bizkaia, Spain.

*ahurtado@neiker.eus

Supplementary Information file

Table S1. Isolate metadata and phenotypic antimicrobial resistance data.

Isolate	Sampling year	Species	Host	ST	CC	AMR phenotypic profile	Minimum inhibitory concentration (mg/L)[c]						
							AMP	CIP	NAL	ERY	GEN	STR	TET
C0574	2014	*C. jejuni*	Dairy cattle	19	21	CIP-NAL	1.5	**8**	**> 64**	< 1	0.5	2	≤ 0.5
C0585	2014	*C. jejuni*	Beef cattle	21	21	CIP-NAL-TET	1	**16**	**> 64**	< 1	0.5	2	**32**
C0596	2015	*C. jejuni*	Beef cattle	21	21	CIP-NAL-TET	0.5	**8**	**> 64**	< 1	0.25	1	**> 64**
C0642	2014	*C. jejuni*	Beef cattle	21	21	CIP-NAL-STR-TET	1.5	**8**	**> 64**	< 1	0.25	**> 16**	**64**
C0459	2014	*C. jejuni*	Dairy cattle	21	21	CIP-NAL-TET	0.5	**16**	**> 64**	< 1	0.5	2	**64**
C0560	2016	*C. jejuni*	Dairy cattle	21	21	CIP-NAL-TET	0.5	**8**	**> 64**	< 1	0.5	2	**64**
C0612	2015	*C. jejuni*	Sheep	21	21	AMP-CIP-NAL-TET	**>256**	**16**	**> 64**	< 1	0.5	2	**16**
C0609	2015	*C. jejuni*	Beef cattle	47	21	CIP-NAL	2	**16**	**> 64**	< 1	≤ 0.12	1	≤ 0.5
C0651	2015	*C. jejuni*	Sheep	50	21	CIP-NAL	1.5	**8**	**> 64**	< 1	0.5	2	≤ 0.5
C0662	2015	*C. jejuni*	Beef cattle	53	21	Non-available	2	NA	NA	NA	NA	NA	NA
C0582	2015	*C. jejuni*	Dairy cattle	53	21	TET	1	≤ 0.12	4	< 1	0.5	2	**64**
C0437	2014	*C. jejuni*	Sheep	883	21	AMP-CIP-NAL-TET	**32**	**16**	**> 64**	< 1	0.5	2	**> 64**
C0622	2015	*C. jejuni*	Beef cattle	3769	21	AMP-CIP-NAL-TET	**32**	**16**	**> 64**	< 1	0.5	2	**4**
C0669	2014	*C. jejuni*	Dairy cattle	3769	21	AMP-CIP-NAL-TET	**64**	**16**	**> 64**	< 1	0.5	2	**4**
C0615	2016	*C. jejuni*	Beef cattle	22	22	CIP-NAL	0.75	**8**	**> 64**	< 1	0.5	2	≤ 0.5
C0575	2015	*C. jejuni*	Dairy cattle	22	22	CIP-NAL	0.75	**8**	**64**	< 1	0.5	2	≤ 0.5
C0882	2019	*C. jejuni*	Dairy cattle	459	42	CIP-NAL-TET	0.5	**8**	**> 64**	< 1	0.5	2	**> 64**
C0426	2014	*C. jejuni*	Dairy cattle	6010	42	TET	0.25	≤ 0.12	4	< 1	0.5	2	**32**
C0584	2014	*C. jejuni*	Beef cattle	6532	42	CIP-NAL-STR-TET	0.25	**8**	**> 64**	< 1	0.5	**> 16**	**64**
C0658	2014	*C. jejuni*	Dairy cattle	6532	42	CIP-NAL-STR-TET	0.38	**8**	**> 64**	< 1	0.5	**> 16**	**64**
C0577	2015	*C. jejuni*	Sheep	6532	42	CIP-NAL-STR-TET	0.38	**8**	**> 64**	< 1	0.5	**> 16**	**64**
C0631	2016	*C. jejuni*	Sheep	45	45	SUSCEPTIBLE	3	≤ 0.12	4	< 1	0.5	2	≤ 0.5
C0424	2014	*C. jejuni*	Sheep	48	48	CIP-NAL	0.5	**16**	**> 64**	< 1	0.5	2	≤ 0.5
C0644	2014	*C. jejuni*	Sheep	10653	49	CIP-NAL	6	**16**	**> 64**	< 1	0.5	2	≤ 0.5
C0576	2015	*C. jejuni*	Dairy cattle	52	52	AMP-CIP-NAL-TET	**16**	**16**	**> 64**	< 1	1	2	**> 64**

C0593	2015	*C. jejuni*	Beef cattle	61	61	TET	1	≤ 0.12	8	< 1	0.5	2	64
C0672	2015	*C. jejuni*	Sheep	122	206	CIP-NAL-TET	2	16	> 64	< 1	0.5	2	64
C0462	2014	*C. jejuni*	Beef cattle	572	206	AMP-CIP-NAL-TET	48	8	> 64	< 1	≤ 0.12	1	64
C0268	2005	*C. jejuni*	Sheep	572	206	AMP-CIP	32	1	16	< 1	0.5	4	1
C0630	2016	*C. jejuni*	Sheep	3188	206	CIP-NAL-TET	1.5	8	64	< 1	0.5	2	32
C0599	2016	*C. jejuni*	Sheep	3188	206	CIP-NAL-TET	1	8	> 64	< 1	0.5	2	64
C0639	2014	*C. jejuni*	Dairy cattle	356	353	AMP-CIP-NAL-TET	>256	8	64	< 1	0.25	1	32
C0614	2016	*C. jejuni*	Beef cattle	5396	353	AMP-CIP-NAL-TET	32	8	> 64	< 1	0.5	1	16
C0667	2014	*C. jejuni*	Dairy cattle	10657	354	AMP-CIP-NAL-TET	> 256	8	> 64	< 1	0.5	1	32
C0661	2015	*C. jejuni*	Beef cattle	10654	460	CIP-NAL-TET	0.25	8	64	< 1	0.25	1	32
C0655	2016	*C. jejuni*	Beef cattle	607	607	AMP-CIP-NAL-TET	> 256	16	> 64	< 1	0.5	2	64
C0623	2015	*C. jejuni*	Beef cattle	1044	658	AMP-TET	96	≤ 0.12	4	< 1	0.5	2	> 64
C0455	2014	*C. jejuni*	Dairy cattle	441	-	AMP-CIP-NAL-TET	48	4	> 64	< 1	0.5	2	64
C0578	2015	*C. jejuni*	Dairy cattle	441	-	AMP-CIP-NAL-TET	64	8	64	< 1	0.5	1	64
C0443	2014	*C. jejuni*	Beef cattle	10652	-	CIP-NAL-TET	1.5	16	> 64	< 1	0.5	2	> 64
C0140	2004	*C. coli*	Dairy cattle	825	828	AMP-CIP-ERY-GEN-NAL-STR-TET	> 256	16	> 64	> 128	> 16	> 16	> 64
C0562	2016	*C. coli*	Sheep	825	828	CIP-NAL-STR-TET	2	16	> 64	< 1	2	> 16	> 64
C0025	2004	*C. coli*	Sheep	825	828	ERY-TET	2	0.25	8	> 128	1	4	> 64
C0663	2015	*C. coli*	Beef cattle	827	828	AMP-CIP-NAL-STR-TET	16	> 16	> 64	< 1	0.5	> 16	> 64
C0535	2014	*C. coli*	Dairy cattle	827	828	STR-TET	8	≤ 0.12	8	< 1	0.5	> 16	> 64
C0551	2015	*C. coli*	Dairy cattle	827	828	CIP-NAL-STR-TET	4	16	> 64	< 1	0.5	> 16	> 64
C0558	2015	*C. coli*	Sheep	827	828	CIP-NAL-TET	4	16	> 64	< 1	0.5	4	> 64
C0561	2016	*C. coli*	Sheep	827	828	CIP-NAL-STR-TET	4	8	64	< 1	1	> 16	> 64
C0601	2016	*C. coli*	Sheep	827	828	STR-TET	8	≤ 0.12	4	< 1	1	8	64
C0563	2016	*C. coli*	Sheep	827	828	CIP-NAL-STR-TET	8	8	> 64	< 1	2	> 16	> 64
C0554	2015	*C. coli*	Sheep	827	828	CIP-NAL-STR-TET	6	16	> 64	< 1	0.5	> 16	> 64
C0444	2014	*C. coli*	Sheep	827	828	SUSCEPTIBLE	8	≤ 0.12	8	< 1	0.5	2	≤ 0.5
C0544	2015	*C. coli*	Beef cattle	1055	828	SUSCEPTIBLE	3	≤ 0.12	4	< 1	1	4	≤ 0.5
C0430	2014	*C. coli*	Dairy cattle	1055	828	STR	3	≤ 0.12	8	< 1	1	8	≤ 0.5

C0553	2015	*C. coli*	Sheep	1177	828	CIP-NAL-STR-TET	2	**8**	**> 64**	< 1	1	**> 16**	**32**
C0545	2015	*C. coli*	Sheep	1590	828	AMP-STR-TET	**> 256**	≤ 0.12	4	< 1	0.5	**> 16**	**32**
C0673	2015	*C. coli*	Sheep	1595	828	AMP-CIP-NAL-STR-TET	**> 256**	**8**	**> 64**	< 1	1	**> 16**	**64**
C0540 [a]	2014	*C. coli*	Dairy cattle	2097	828	AMP-CIP-ERY-NAL-TET	**> 256**	**16**	**> 64**	**> 128**	1	4	**> 64**
C0541 [a]	2014	*C. coli*	Dairy cattle	2097	828	AMP-CIP-ERY-GEN-NAL-STR-TET	**> 256**	**16**	**> 64**	**> 128**	**> 16**	**> 16**	**> 64**
C0680	2015	*C. coli*	Dairy cattle	2097	828	AMP-CIP-ERY-GEN-NAL-STR-TET	**> 256**	**> 16**	**> 64**	**> 128**	**> 16**	**> 16**	**> 64**
C0677 [b]	2016	*C. coli*	Dairy cattle	2097	828	AMP-CIP-ERY-GEN-NAL-STR-TET	**> 256**	**> 16**	**> 64**	**> 128**	**> 16**	**> 16**	**> 64**
C0678 [b]	2016	*C. coli*	Dairy cattle	2097	828	AMP-CIP-ERY-GEN-NAL-STR-TET	**64**	**> 16**	**> 64**	**> 128**	**> 16**	**> 16**	**> 64**
C0566	2016	*C. coli*	Dairy cattle	2097	828	AMP-CIP-GEN-NAL-STR-TET	**96**	**16**	**> 64**	< 1	**> 16**	**> 16**	**> 64**
C0539	2014	*C. coli*	Beef cattle	5111	828	CIP-NAL-TET	6	**8**	**64**	< 1	0.5	2	**> 64**
C0428	2014	*C. coli*	Sheep	5380	828	CIP-NAL-TET	4	**16**	**> 64**	< 1	0.5	2	**> 64**
C0564	2016	*C. coli*	Dairy cattle	6510	828	AMP-CIP-NAL-STR-TET	**> 256**	**> 16**	**64**	< 1	0.5	**> 16**	**> 64**
C0538	2014	*C. coli*	Dairy cattle	6775	828	CIP-NAL-TET	1.5	**16**	**> 64**	< 1	0.5	2	**> 64**
C0548	2015	*C. coli*	Beef cattle	8313	828	CIP-NAL-STR-TET	4	**> 16**	**> 64**	< 1	0.5	**16**	**> 64**
C0546	2015	*C. coli*	Beef cattle	8857	-	CIP-NAL-STR-TET	2	**> 16**	**> 64**	< 1	1	**> 16**	**> 64**
C0559	2016	*C. coli*	Sheep	8857	-	CIP-NAL-STR	6	**> 16**	**> 64**	< 1	0.5	**> 16**	≤ 0.5

[a, b], Isolates with the same superscript were isolated in the same farm.

[c], values above the ECOFF are indicated in bold red. Ampicillin, AMP >8 mg/L; ciprofloxacin, CIP >0.5mg/L; nalidixic acid, NAL >16 mg/L; erythromycin, ERY >8mg/L (*C. coli*) and >4 mg/L (*C. jejuni*); gentamicin, GEN >2mg/L; streptomycin, STR >4 mg/L; tetracycline, TET >2 mg/L (*C. coli*) and >1 mg/L (*C. jejuni*). Results for CIP, NAL, ERY, GEN, STR and TET were already published (Ocejo et al., 2019); results for AMP are part of this study.

Table S2. Overview of sequencing raw data and assembly for each sample.

Raw Reads Stats				Assembly Stats				
Isolate	Species	Total reads	Coverage X)	N contigs	Largest Contig	N50	Genome Length (bp)	%GC
C0268	*C. jejuni*	12934244	1173	73	226969	154175	1653564	30.5
C0424	*C. jejuni*	10540130	958	41	412558	197793	1650143	30.5
C0426	*C. jejuni*	12245504	1074	39	375606	187262	1709280	30.5
C0437	*C. jejuni*	14300506	1248	66	546850	153956	1717905	30.5
C0443	*C. jejuni*	11988856	1060	88	388021	113547	1696244	30.5
C0455	*C. jejuni*	13069366	1166	52	581808	156346	1680588	30.5
C0459	*C. jejuni*	10414200	931	60	410914	155089	1676447	30.5
C0462	*C. jejuni*	12836158	1092	32	772278	191279	1762483	30.4
C0560	*C. jejuni*	17067854	1520	79	230397	154524	1683741	30.5
C0574	*C. jejuni*	10859498	981	36	402450	153949	1659726	30.4
C0575	*C. jejuni*	11498010	1042	34	235461	184884	1654883	30.5
C0576	*C. jejuni*	10232652	907	37	358497	211364	1691424	30.4
C0577	*C. jejuni*	13380738	1246	43	336369	212864	1609799	30.6
C0578	*C. jejuni*	12901696	1152	54	416441	156346	1678853	30.5
C0582	*C. jejuni*	9723978	895	43	556545	298216	1628019	30.6
C0584	*C. jejuni*	13908460	1294	53	336160	212864	1612235	30.6
C0585	*C. jejuni*	15730320	1376	62	406922	140820	1713798	30.4
C0593	*C. jejuni*	11641514	1049	46	406509	232929	1663253	30.4
C0596	*C. jejuni*	10812544	933	63	409211	139199	1738344	30.5
C0599	*C. jejuni*	11557242	981	78	576024	105433	1766292	30.4
C0609	*C. jejuni*	15310196	1374	50	500065	255847	1670710	30.5
C0612	*C. jejuni*	11351426	974	47	407161	154480	1747161	30.4
C0614	*C. jejuni*	12462898	1096	95	314832	188509	1705294	30.5
C0615	*C. jejuni*	10042304	929	47	230341	151286	1621057	30.5
C0622	*C. jejuni*	10893806	984	35	402447	159911	1659826	30.5
C0623	*C. jejuni*	11680350	1054	43	404577	215773	1661697	30.5
C0630	*C. jejuni*	9920180	844	68	403655	108264	1761096	30.4
C0631	*C. jejuni*	11308098	1015	55	307379	204078	1670764	30.5
C0639	*C. jejuni*	12880436	1119	47	334575	183859	1725830	30.3
C0642	*C. jejuni*	11959798	1032	51	590192	104959	1737520	30.5
C0644	*C. jejuni*	12786404	1157	42	546255	289216	1656869	30.5
C0651	*C. jejuni*	12295578	1130	44	645526	188193	1632065	30.5
C0655	*C. jejuni*	8773988	702	176	259598	91985	1872331	30.1
C0658	*C. jejuni*	13181088	1229	43	319610	186698	1608075	30.6
C0661	*C. jejuni*	12878332	1059	83	297925	139572	1823462	30.2
C0662	*C. jejuni*	13719044	1237	49	419090	180719	1663018	30.4
C0667	*C. jejuni*	15428792	1333	62	339673	208934	1736132	30.3
C0669	*C. jejuni*	15009370	1354	44	567135	159910	1661932	30.5
C0672	*C. jejuni*	6656444	593	34	440430	160973	1682240	30.5

C0882	*C. jejuni*	14705740	1345	49	224960	150692	1639337	30.5
C0025	*C. coli*	10122034	917	49	446779	172397	1654969	31.4
C0140	*C. coli*	10979218	983	37	596293	265693	1673787	31.5
C0428	*C. coli*	16828292	1530	23	815050	255274	1649310	31.4
C0430	*C. coli*	11474124	1043	32	596292	395173	1649217	31.5
C0444	*C. coli*	12233762	1097	52	598846	266139	1672213	31.4
C0535	*C. coli*	12342818	1108	24	596148	403503	1669892	31.4
C0538	*C. coli*	16454080	1441	63	285352	210646	1711832	31.3
C0539	*C. coli*	15546954	1319	88	278640	130395	1767284	31.4
C0540	*C. coli*	12493926	1044	38	281441	162622	1793431	31.3
C0541	*C. coli*	13483176	1110	96	281441	147642	1820453	31.4
C0544	*C. coli*	16741992	1523	36	596291	265706	1648196	31.5
C0545	*C. coli*	14110464	1277	51	380940	215171	1656510	31.4
C0546	*C. coli*	10837624	914	60	417840	215914	1777565	31.3
C0548	*C. coli*	18585354	1675	34	396807	196506	1663543	31.4
C0551	*C. coli*	9191142	811	31	590028	402358	1698856	31.4
C0553	*C. coli*	12929314	1062	51	313038	163968	1825109	31.2
C0554	*C. coli*	16326156	1437	46	596396	265702	1704116	31.4
C0558	*C. coli*	14428070	1294	36	827468	265684	1671537	31.5
C0559	*C. coli*	14532030	1248	71	379836	264568	1746486	31.2
C0561	*C. coli*	14042064	1256	42	442254	229068	1676468	31.4
C0562	*C. coli*	11966072	1075	32	396508	181044	1669298	31.4
C0563	*C. coli*	12675758	1136	34	380950	229088	1673402	31.4
C0564	*C. coli*	10813858	953	43	443471	226674	1700557	31.4
C0566	*C. coli*	11977774	1030	51	281500	162622	1743404	31.4
C0601	*C. coli*	11763842	1056	31	585237	265699	1670365	31.4
C0663	*C. coli*	14313310	1278	43	594415	265686	1679325	31.5
C0673	*C. coli*	9951316	867	35	435153	218424	1721131	31.4
C0677	*C. coli*	15002976	1290	58	284762	163975	1744337	31.4
C0678	*C. coli*	17061266	1471	38	284764	163976	1739270	31.4
C0680	*C. coli*	10690064	862	48	280581	139165	1859162	31.2

Figure S1. Circular map of the pTet (type-1) plasmid of *C. jejuni* isolates C0599, C0585, C0612 and C063, along with plasmid pFB1TET of *C. coli* strain FB1 (Acc. Nr. CP011017) as an outer circle.

The BLAST Ring Image Generator (BRIG) software [1] was used for comparison analysis of plasmids and image generation.

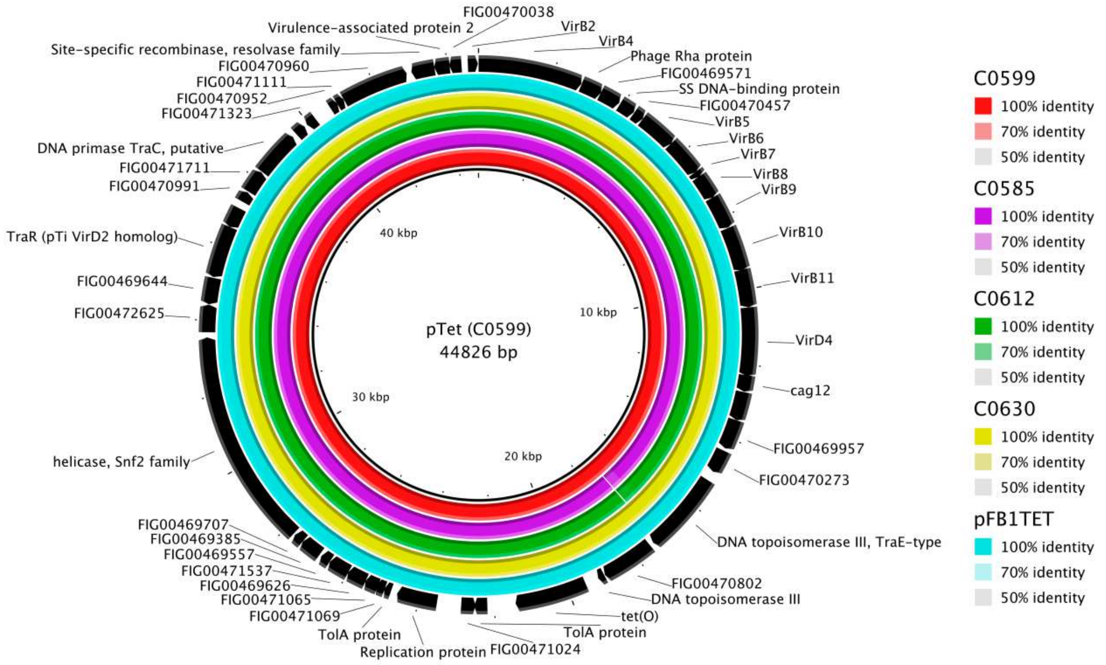

References

1. Alikhan, N. F., Petty, N. K., Ben Zakour, N. L. & Beatson, S. A. BLAST Ring Image Generator (BRIG): Simple prokaryote genome comparisons. *BMC Genomics* **12**, 402 (2011).
